# Geographic Distribution of Florida's Orthopedic Surgery Workforce: A County-Level Analysis

**DOI:** 10.7759/cureus.115788

**Published:** 2026-09-04

**Authors:** Shane Burke, Alan Wu, Shervin Eskandari, Dev Shah, Cole Kramer, Porter Young

**Affiliations:** 1 Orthopedic Surgery, University of Central Florida, Orlando, USA; 2 Orthopedic Surgery, Augusta University at Medical College of Georgia, Augusta, USA; 3 Orthopedic Surgery, University of South Florida, Tampa, USA; 4 Orthopedic Surgery and Rehabilitation, University of Florida College of Medicine-Jacksonville, Jacksonville, USA

**Keywords:** geographic access, healthcare equity, orthopedic surgery, rural-urban disparities, workforce distribution

## Abstract

Background: As Florida's population growth surges, access to orthopedic care is increasingly important, yet statewide geographic accessibility remains underexplored. This study aims to conduct a county-level analysis of the distribution and availability of orthopedic surgeons serving Florida's rural and urban populations.

Methods: Orthopedic surgeons licensed to practice in Florida were identified using the Florida Department of Health Practitioner Profile database, including primary clinical practice address and residency training state. Counties were categorized as metropolitan (Rural-Urban Continuum Codes {RUCC} 1-3) or non-metropolitan (RUCC 4-9) using 2023 RUCC designations. County demographic and geographic variables were obtained from the 2020 US Census Bureau.

Results: A total of 2,117 orthopedic surgeons were identified across Florida's 67 counties (45 metropolitan and 22 non-metropolitan), spanning 53,654 square miles. In total, 19 counties (28.4%) lacked a practicing orthopedic surgeon; 14 (73.7%) were classified as non-metropolitan. Metropolitan counties accounted for 2,098 (99.1%) surgeons, with the most urban counties (RUCC 1) exhibiting the greatest surgeon density and lowest average travel burden. The average surgeon density in metropolitan counties was 8.33 per 100,000 residents with an average travel burden of 8.24 miles, compared to a density of 1.79 surgeons per 100,000 residents and a longer average travel burden of 21.03 miles in non-metropolitan counties (p < 0.001).

Conclusions: Substantial rural-urban disparities exist in Florida's orthopedic workforce, with non-metropolitan counties facing pronounced shortages and longer travel distances for care. These findings support region-specific workforce planning and physician retention strategies to improve geographic equity as statewide demand continues to rise.

## Introduction

Florida's rapid population growth has placed increasing strain on the state's healthcare infrastructure, particularly in subspecialty fields such as orthopedic surgery [1]. From 2010 to 2019, Florida's population grew by over 2.6 million residents, rising from 18.8 to 21.5 million, housing the third-highest percentage of people over the age of 65 in the country [2,3]. When considering the aging population of the United States, and Florida in particular, it is important to have orthopedic surgeons available to manage the burden of musculoskeletal conditions that disproportionately affect older adults [4,5]. Nationally, orthopedic surgeons are overwhelmingly concentrated in urban areas, with the proportion practicing in urban counties increasing from 77% in 2000 to 93% in 2019 [6]. Although primary care physicians can treat simple musculoskeletal complaints, complex conditions such as tendinopathies, enthesopathies, and fractures more often require specialized surgical expertise [7].

Rural-urban disparities in orthopedic care have persisted over the past decade and may continue to widen in the future, with a 0.9% decrease in the proportion of rural orthopedic surgeons from 2013 to 2018 [8]. Although there is no state-specific study limited to Florida that focuses on orthopedic surgeon distribution, national results are highly indicative of this state-level pattern. As the second fastest-growing state by numeric growth, it is essential to evaluate and address the distribution of orthopedic surgeons in Florida to ensure that expanding populations have suitable access to care amid widening rural-urban disparities.

Improving access to healthcare in rural areas is critical to address the longitudinal disparities in health outcomes. It was discovered that rural patients with lower incomes who suffered hip fractures were 32% more likely to suffer from fatal complications than urban patients with hip fractures [9]. Furthermore, the number of specific surgeries such as meniscectomies and meniscal repairs, as well as patients who received allograft during meniscal procedures, was disproportionately concentrated in urban areas [10].

Effective policy development, strategic intervention, and equitable resource allocation depend on a holistic understanding of the geographic distribution of the Florida orthopedic workforce. Therefore, our study assessed county-level surgeon density and travel burden to characterize the spatial distribution of orthopedic surgeons across the state and evaluate access disparities between urban and rural communities.

## Materials and methods

A cross-sectional analysis of the Florida Department of Health Practitioner Profile database was conducted to identify the geographic distribution of actively practicing orthopedic surgeons in Florida [11]. Physicians in the state are required to report various credentials on this platform, which are then verified by the Florida Department of Medical Quality Assurance. This publicly accessible database was used to gather information on all physicians holding active orthopedic surgery licenses, including their primary office location and, where applicable, the state in which they completed their residency training. The identities of the surgeons and their practice locations were further confirmed through publicly available business websites. Records were de-duplicated by state license number to ensure that each surgeon was represented only once. Additionally, where a surgeon practiced at more than one location, the clinical address in the database was used. Surgeons listed in databases indicating practice outside of Florida, or with licensure statuses such as revoked, delinquent, null and void, voluntarily relinquished, or active but not practicing, were excluded from the analysis.

County-level data, including overall and specific population estimates by rurality, median household income, and county coverage in square miles, were obtained from the US Census Bureau and the Florida Legislature, Office of Economic and Demographic Research [12,13]. Additionally, counties were classified as metropolitan or non-metropolitan based on the 2023 Rural-Urban Continuum Codes (RUCC) [14]. This system stratifies metropolitan counties based on population estimates within the metropolitan (metro) areas while classifying non-metropolitan (non-metro) counties based on their proximity to metropolitan areas and level of urbanization. Counties are assigned one of nine possible codes. Codes 1-3 are categorized as metropolitan counties: 1, metropolitan areas with a minimum population of one million; 2, metropolitan areas with a population between 250,000 and one million; and 3, metropolitan areas with a maximum population of 250,000. Codes 4-9 are categorized as non-metropolitan codes, defined as follows: 4, metro-adjacent areas with an urban population of 20,000 or more; 5, counties with an urban population of 20,000 or more that are not adjacent to a metropolitan county; 6, metro-adjacent areas with an urban population of 5,000-20,000; 7, counties with an urban population of 5,000-20,000 that are not adjacent to a metropolitan area; 8, metro-adjacent counties with an urban population of fewer than 5,000; and 9, areas with an urban population of fewer than 5,000 that are not adjacent to a metropolitan area.

County-specific measures, including the number of orthopedic surgeons per 100,000 residents and the number of orthopedic surgeons per 100 square miles, were calculated and compared across the state. The travel burden for individuals within each county was assessed using the linear (haversine) distance from the coordinates of the county centroid to the coordinates of the nearest orthopedic surgery practice derived from addresses. County centroids were defined using the US Census Bureau's TIGER/Line internal-point coordinates for each county. Straight-line distance was used to provide a consistent, reproducible measure across all 67 counties, independent of any particular routing engine or road dataset, which could vary output. Statistical analysis was conducted using SPSS v29.0.0.0 (IBM Corp., Armonk, NY). The Kruskal-Wallis test was performed to analyze data stratified by Rural-Urban Continuum Code, and the Mann-Whitney U test was used to analyze county-specific data classified as either metropolitan or non-metropolitan. In each case, statistical significance was determined with a threshold of p < 0.05. For the Kruskal-Wallis tests, the tie-corrected H statistic is reported together with its degrees of freedom (k - 1 = 6, corresponding to the seven Rural-Urban Continuum Code groups present in Florida); for the Mann-Whitney U tests, the U statistic is reported together with the standardized Z value. Effect sizes were calculated as epsilon-squared (ε² = H/(N - 1)) for the Kruskal-Wallis tests and as r (= |Z|/ÖN) for the Mann-Whitney U tests. Where a Kruskal-Wallis test was significant, pairwise comparisons were performed using Dunn's test with Bonferroni correction for multiple comparisons.

## Results

A total of 2,117 orthopedic surgeons were identified in this study, serving Florida's 21,538,187 residents across 53,654 square miles and 67 counties. Among the 2,119 surgeons with medical school type recorded, 1,873 (88.4%) graduated from allopathic schools, and 246 (11.6%) graduated from osteopathic schools. Of the 1,803 surgeons with a recorded residency training location, the most common were Florida (340; 18.9%), New York (291; 16.1%), and Pennsylvania (116; 6.4%); 67 (3.7%) trained outside the United States.

Of the total Florida population, 1,823,381 (8.5%) resided in rural areas. The statewide average (standard deviation {SD}) was 6.18 (5.70) orthopedic surgeons per 100,000 residents and 3.73 (6.69) orthopedic surgeons per 100 square miles. The average (SD) travel burden to the nearest orthopedic surgeon across all counties was 12.37 (10.95) miles. Additionally, the average median household income was $54,472.42 ($9,488.47) statewide.

**Figure 1:**
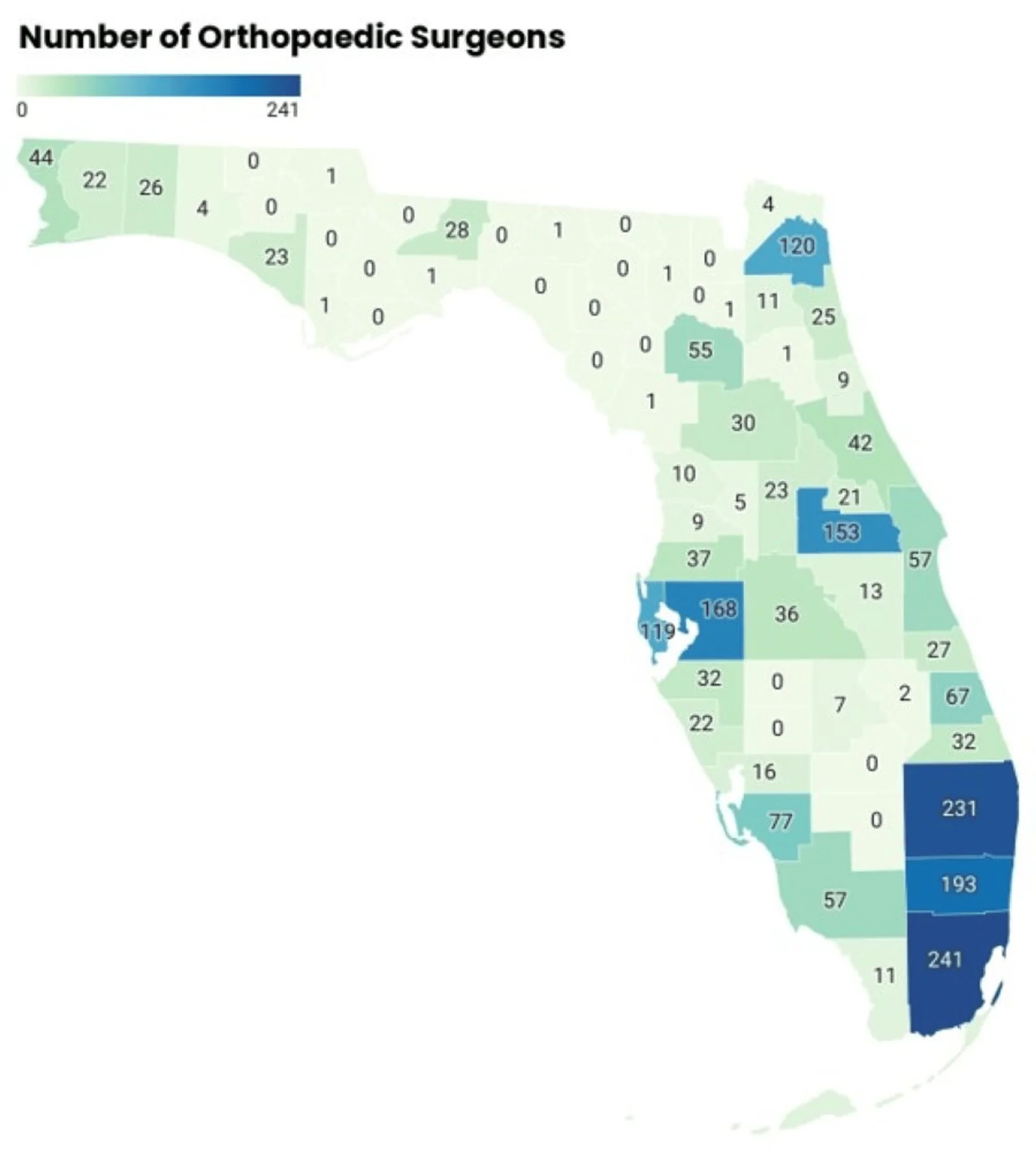
County-level number of orthopedic surgeons in Florida.

**Figure 2:**
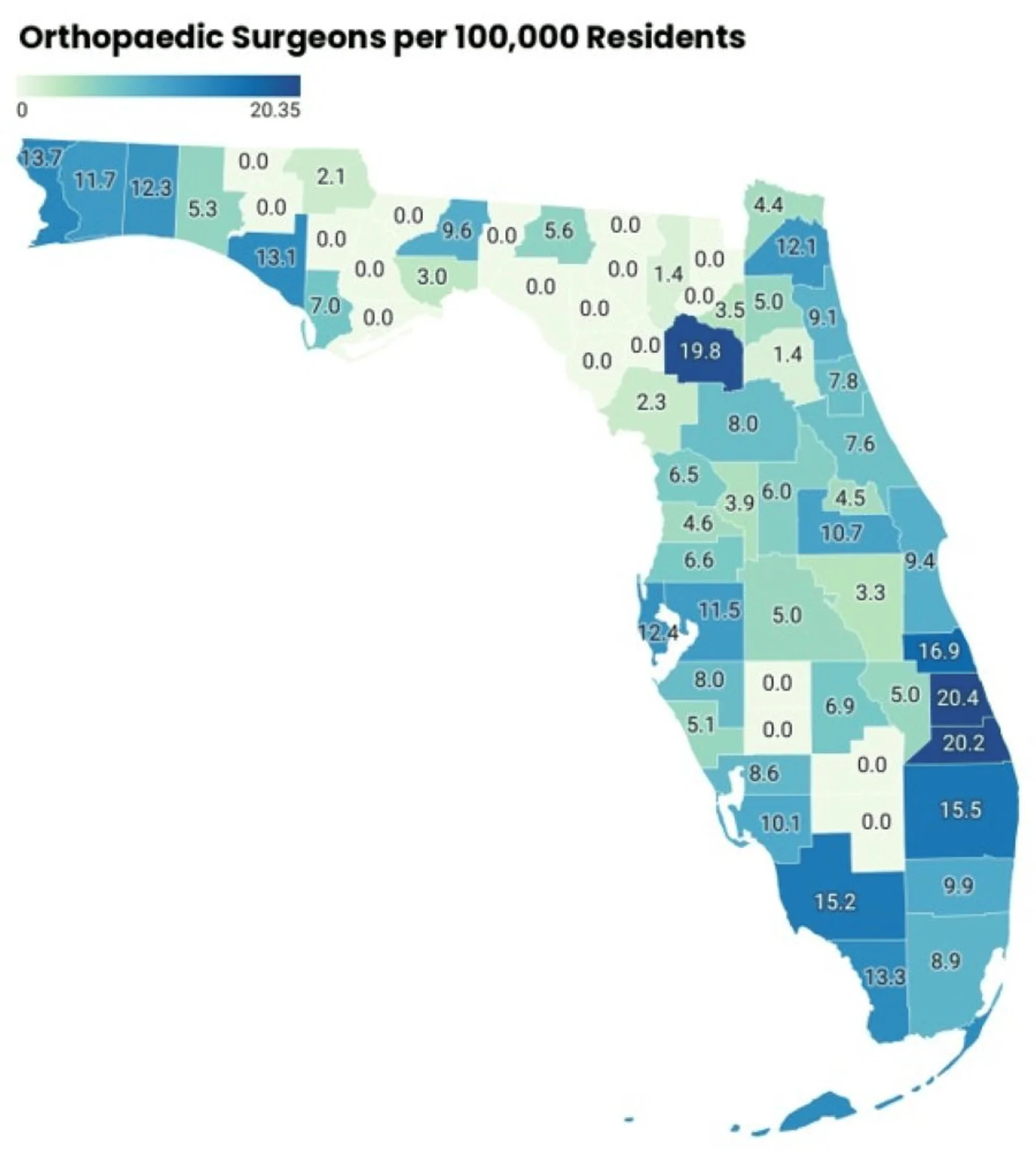
Distribution of the Florida orthopedic workforce as represented by the number of orthopedic surgeons per 100,000 people.

Metropolitan counties (n = 45) comprised 96.9% of the state's total population, while non-metropolitan counties (n = 22) represented 3.1%. Among all counties, 19 (28.4%) did not have a practicing orthopedic surgeon, and 14 (73.7%) of these were classified as non-metropolitan. Most orthopedic surgeons (n = 2,098; 99.1%) practiced in metropolitan counties, with only 19 (0.9%) in non-metropolitan counties. The distribution of the Florida orthopedic workforce is displayed in Figure 1 and Figure 2.

Counties classified as code 1 (most urbanized) had the highest number of orthopedic surgeons (n = 1,366), the greatest surgeon density (mean {SD} = 7.99 {4.19} per 100,000 residents), and the lowest average travel burden (mean {SD} = 7.63 {7.30} miles). In contrast, the most rural counties (codes 8 and 9) collectively had only two orthopedic surgeons, a combined population of 153,148, and the highest average travel burden (23.39 miles and 30.76 miles, respectively). A detailed breakdown of county-level orthopedic surgeon availability and access by rural-urban classification is provided in Table 1. Surgeon supply and access varied sharply across the rurality gradient: Kruskal-Wallis tests, reported in Table 2, showed large effects for both density measures, most notably surgeons per 100 square miles (H(6) = 33.36; p < 0.001; ε² = 0.51), and a large effect for travel burden as well (H(6) = 18.07; p = 0.006; ε² = 0.27). Dunn's post hoc comparisons (Table 3) localized these differences to contrasts between the most urban (RUCC 1-2) and the most rural (RUCC 6 and 8) groups, consistent with an access gradient that becomes statistically distinct only at the extremes of the rurality scale.

**Table 1:** County-level orthopedic surgeon supply and travel burden by Rural-Urban Continuum Code classification.

Rural-Urban Classification	Counties	Total Population	Rural Census	Rural, %	Total Square Miles	Total Number of Orthopedic Surgeons	Average Orthopedic Surgeons per 100k	Average Orthopedic Surgeons per 100 Square Miles	Travel Burden (mi)
1	16	13,470,557	460,911	3.4	14,935	1,366	7.99 (4.19)	9.49 (11.16)	7.63 (7.30)
2	22	6,477,132	767,691	11.9	18,894	644	8.68 (6.25)	3.70 (3.28)	8.24 (7.02)
3	7	931,999	160,473	17.2	4,684	88	7.98 (5.63)	2.01 (1.81)	9.16 (8.27)
4	5	305,156	137,232	45	4,434	15	4.22 (5.39)	0.33 (0.45)	19.65 (13.61)
6	6	200,195	146,025	72.9	4,218	2	0.94 (1.53)	0.07 (0.14)	16.39 (12.70)
8	10	144,922	142,823	98.6	5,946	2	1.26 (2.68)	0.03 (0.07)	23.39 (12.48)
9	1	8,226	8,226	100	543	0	0.00 (0.00)	0.00 (0.00)	30.76 (0.00)
Total	67	21,538,187	1,823,381	8.5	53,654	2,117	6.18 (5.70)	3.73 (6.69)	12.37 (10.95)

**Table 2:** Kruskal-Wallis test results for orthopedic surgeon density and travel burden across Rural-Urban Continuum Code groups. k = 7 groups, df = 6, and N = 67. H is tie-corrected. ε² = H/(N - 1); η² = (H - k + 1)/(N - k), shown for reference. df: degrees of freedom

Variable	H	df	p	ε²	η²
Orthopedic surgeons per 100,000 residents	24.31	6	<0.001	0.37	0.31
Orthopedic surgeons per 100 square miles	33.36	6	<0.001	0.51	0.46
Travel burden to nearest surgeon (miles)	18.07	6	0.006	0.27	0.20

**Table 3:** Significant Dunn's post hoc pairwise comparisons following Kruskal-Wallis tests, with Bonferroni- and Holm-adjusted p values. RUCC: Rural-Urban Continuum Code

	Comparison	p (Bonferroni)	p (Holm)
Orthopedic surgeons per 100,000 residents	RUCC 1 versus 8	0.015	0.014
	RUCC 2 versus 6	0.044	0.040
	RUCC 2 versus 8	0.007	0.007
Orthopedic surgeons per 100 square miles	RUCC 1 versus 6	0.005	0.004
	RUCC 1 versus 8	<0.001	<0.001
	RUCC 2 versus 8	0.003	0.003
Travel burden to nearest surgeon (miles)	RUCC 1 versus 8	0.034	0.034
	RUCC 2 versus 8	0.034	0.034

On average, there was a significantly greater number of orthopedic surgeons per 100,000 residents serving metropolitan counties compared to non-metropolitan counties (mean {SD} = 8.33 {5.39} versus 1.79 {3.33}; p < 0.001). Metropolitan counties also had a significantly lower average travel burden than non-metropolitan counties (mean {SD} = 8.24 miles {7.16} versus 21.03 miles {12.42}; p < 0.001) and higher median household incomes (mean {SD} = $57,528.00 {9,488.47} versus $43,774.09 {8,284.82}; p < 0.001). There were also significantly greater orthopedic surgeons per 100 square miles in metropolitan counties compared to non-metropolitan counties (mean {SD} = 5.50 {7.57} versus 0.11 {0.25}; p < 0.001). Every workforce and access measure favored metropolitan counties by a wide margin, with the largest effects for surgeons per county and per 100 square miles (both r = 0.66); county land area was the only measure that did not differ significantly between groups (p = 0.251; r = 0.14), indicating that these disparities reflect workforce distribution rather than differences in county size. Comparison of key demographic and access variables between metropolitan and non-metropolitan counties is summarized in Table 4.

**Table 4:** Comparison of orthopedic surgeon density, access, and travel burden between metropolitan and non-metropolitan counties. Data are presented as mean (standard deviation) when applicable.

	Metropolitan	Non-metropolitan	U	W	Z	r	P value
Counties	45	22	-	-	-	-	-
Total population	20,879,688	658,499	-	-	-	-	-
Rural census	1,389,075	434,306	-	-	-	-	-
Rural, %	6.7	66	-	-	-	-	-
Total square miles	38,512	15,141	-	-	-	-	-
Total orthopedic surgeons	2,098	19	-	-	-	-	-
Average county size (mi²)	855.8 (354.0)	688.3 (301.2)	409.0	1,616.0	1.14	0.14	0.25
Average percent rural	23.8 (21.8)	78.6 (19.3)	107.0	1,142.0	-5.20	0.64	<0.001
Average median household income	$57,528 ($7,749)	$43,774 ($8,327)	123.0	1,902.0	4.96	0.61	<0.001
Average orthopedic surgeons per county	46.6 (61.5)	0.9 (2.3)	92.5	1,932.5	5.43	0.66	<0.001
Average orthopedic surgeons/100k	8.33 (5.39)	1.79 (3.33)	146.0	1,879.0	4.71	0.57	<0.001
Average orthopedic surgeons/100 mi²	5.50 (7.57)	0.11 (0.25)	92.0	1,933.0	5.44	0.66	<0.001
Average travel burden (mi)	8.2 (7.16)	21.0 (12.42)	198.0	1,233.0	-3.96	0.48	<0.001

## Discussion

Current projections indicate a 10% increase in demand for orthopedic specialty care by the year 2036; however, the corresponding supply is not expected to keep pace. While the nationwide orthopedic workforce is expected to decline by 2%, the deficit is anticipated to be most pronounced in non-metropolitan counties, with workforce adequacy as low as 52% [15]. Some studies estimate the orthopedic surgeon shortage to be as high as 34.3% by 2040 [16]. This is especially relevant to the state of Florida, given its rapid growth in both metropolitan and non-metropolitan regions in recent decades. It is also essential to consider this in the context of the well-described demographics that define non-metropolitan communities nationwide, as well as within the state. Long-standing nationwide labor market analyses indicate that these regions contain a greater proportion of individuals who hold jobs in manual labor, manufacturing, agriculture, and other physically demanding industries [17]. Florida, specifically, is home to 15,156 square miles of farmland and provides an estimated 1,529,864 jobs to its residents across the agriculture, natural resource, and food manufacturing industries [18]. The workers who are the backbone of these sectors are often characterized by lower educational attainment, lower private insurance enrollment, and lower median incomes compared to those in urban regions. Naturally, these blue-collar occupations also carry a greater risk of workplace-related orthopedic injuries or degenerative joint disease, which may require evaluation or even intervention [19]. Despite this, these areas have been consistently characterized by low densities of orthopedic surgeons, ultimately leading to increased trauma-related mortality and greater reliance on emergency departments, subjecting patients potentially to longer hospital stays, higher expenditures, and potentially poorer outcomes on a national scale [9,19-21].

The results of our study indicate an imbalance in the distribution of orthopedic surgeons across Florida's rural and urban communities, with 99.1% of specialists concentrated in metropolitan areas. This county-level variation highlights a substantial underrepresentation of orthopedic specialists in Florida's non-metropolitan communities. Notably, 19 counties (28.4%) have no practicing orthopedic surgeons, of which 14 counties were classified as non-metro by the Rural-Urban Continuum Code. Moreover, 94.9% of Florida's orthopedic surgeon workforce is concentrated in counties denoted by RUCC codes 1 and 2 (most urban), even though these counties represent slightly over 50% of the state's total and land area in square miles. These observations, however, align with previous nationwide inquiries, which have noted that 51% of counties nationwide lack an orthopedic specialist, and approximately 93% of the orthopedic workforce is localized to metropolitan areas, up from 77% in previous decades [6]. A state-level analysis in Kansas similarly concluded that while the orthopedic workforce increased by 48% from 2009 to 2019, the increase was disproportionately concentrated in the state's non-rural counties, which experienced a growth rate of 78% [22].

Overall, Florida averages 6.18 orthopedic surgeons per 100,000 residents, lower than the national and South Atlantic census division averages of 9.25 and 8.96 orthopedic surgeons per 100,000 residents in 2018 [23]. Differences also persist at the county level when stratified by population size and land area (metro versus non-metro). Specifically, Florida metro counties have 8.33 surgeons per 100,000 residents and 5.50 surgeons per 100 square miles compared to 1.79 surgeons per 100,000 residents and 0.11 surgeons per 100 square miles in non-metro counties (p < 0.001). While the distribution of surgeons may follow the distribution of Florida's population (96.9% metropolitan), a gap remains when geographic access is considered, as only 19 surgeons can be found in a land area nearly a third of the total square mileage of the state. Additionally, when compared to nationwide averages of 22 and two surgeons per 100,000 residents in metro and non-metro counties, Florida falls significantly short of the average metropolitan surgeon density [6]. Albeit these national benchmarks come from different data sources and time periods, they should be interpreted as an approximate value rather than directly equivalent. This underperformance is alarming, as Florida is the third-largest state by population and consistently leads the nation in percentage of population growth, reaching as high as 1.9% in the years 2022-2023. Four of the five fastest-growing metropolitan areas in the country are located in Florida, which also suggests a potential need for the realignment of the orthopedic workforce on a statewide scale [24,25].

Differences in travel burden were also apparent across metropolitan and non-metropolitan counties. On average, the travel distance to an orthopedic surgeon in metropolitan counties was 8.2 miles versus 21.0 miles in non-metropolitan counties (p < 0.001). This number only reflects geographic proximity, as it likely underestimates the barriers and challenges that one might face in a rural community, such as limited public transportation, inadequate roadway infrastructure, or even limited internet access [26-28]. When considered, these factors create a logistically complex scenario for the patient in need. In combination with the added time and expenses of extended travel, it may even deter a patient in need from considering a trip to the specialist in the first place.

Interestingly, Alachua, Martin, St. Lucie, and Indian River counties have the highest surgeon density, although they are not the most metropolitan by RUCC code or the most densely populated. In contrast, Florida's largest and most urbanized counties, such as Miami-Dade, Broward, Palm Beach, Hillsborough, Duval, and Orange counties, have lower surgeon population ratios despite having the highest total number of physicians. The fact that these densities are overwhelmed by the massive general population could pose a threat to the specialty infrastructure if demand for care continues to outpace capacity. Nevertheless, the aforementioned counties have a significantly higher average surgeon density than Florida non-metropolitan counties, supporting the theory that academic institutions or sociodemographic factors, such as median income (p < 0.001), influence physician location of practice, although the problem may be more complex in Florida.

In light of these findings, the solution is rather complex. Historically, strategies such as teleconsultation and remote patient monitoring have aimed to reduce both direct and indirect costs to patients. Nonetheless, access to broadband internet should be considered in non-metropolitan areas. Results of a national study indicated that 14% of US counties both lack an orthopedic surgeon and have less than 50% broadband coverage [29]. Certainly, telehealth has the potential to expand the reach of the surgical workforce; however, it does not wholly resolve the issue, especially when considering surgical needs and digital infrastructure. Similarly, another solution common in the literature is the expansion of workforce development. However, Florida is already home to 46 post-graduate year 1 (PGY-1) residency positions in orthopedic surgery, making it the seventh-largest training pool in the country. Despite this, only 18.9% of physicians practicing in Florida were trained in the state. This is relatively unsurprising considering 2025 reports by the Association of American Medical Colleges that 44.2% of orthopedic residents remain in the state in which they trained, with the majority (55.8%) practicing elsewhere [30]. Together, these findings indicate that there is no one-size-fits-all approach to the issue. Hence, the purpose of this study was to elucidate the current state of Florida's orthopedic workforce to promote further dialogue around the implications for future workforce planning, which could pose a threat to the statewide infrastructure in the long term.

This study, although comprehensive, should be interpreted in light of its limitations. While this study provides a thorough analysis of the orthopedic workforce in Florida, its primary focus was on physicians actively practicing with an MD or DO degree. Consequently, advanced practice providers (APPs), physician assistants, physical therapists, and other healthcare providers were excluded, despite their vital role in the care of an orthopedic patient. National data on the county-level distribution of APPs in orthopedic surgery suggests that the density of APPs is higher than that of orthopedic surgeons, 18 per 100,000 versus eight per 100,000, although they are unlikely to be in counties without practicing surgeons [31]. Thus, the analysis may slightly underestimate the true distribution when considering these individuals. Additionally, the accuracy of the data was confined to physician reports within the Florida Department of Health Practitioner Profile database. The primary practice address provided by physicians was analyzed, albeit many physicians may operate at multiple locations, which could potentially enhance access to service in some areas while overestimating it in others. Similarly, physician demand was not considered in county-level distribution analysis, which could be a driving force behind where physicians choose to operate. Lastly, to maintain consistency in geographic analysis, the travel burden within each county was estimated using the linear distance from the county's centroid, rather than actual driving distance. Straight-line distance was chosen over road-network distance or travel time to keep this measure consistent and reproducible across all counties, though it does not capture road infrastructure or travel time. This approach may have resulted in either an underestimation or an overestimation of the true travel burden from the county centroid to the nearest orthopedic practice. Geographic proximity also does not fully capture patient access, which may additionally depend on wait times, insurance acceptance, referral pathways, and a practice's operative capacity, which were not assessed in this study.

## Conclusions

Florida shows evident rural-urban disparities in orthopedic care, with rural counties facing a shortage of orthopedic surgeons. Relative to urban areas, these counties have significantly fewer surgeons per 100,000 residents and per 100 square miles, coupled with markedly longer travel times to the nearest provider. It is also worth noting that even though urban counties in Florida have a large volume of orthopedic surgeons, the high population density may place a heavy demand on these providers, with the potential to overwhelm available resources.
